# p38 mitogen-activated protein kinase drives senescence in CD4^+^ T lymphocytes and increases their pathological potential

**DOI:** 10.1186/s12979-025-00526-8

**Published:** 2025-07-15

**Authors:** Luis González-Osuna, Sandra Yasuyo Fukada, María Paz Hernández-Cáceres, Patricia Luz-Crawford, Cristian Cortez, Carolina Rojas, Paola Carvajal, Alfredo Sierra-Cristancho, Rolando Vernal

**Affiliations:** 1 Periodontal Biology Laboratory, Faculty of Dentistry, Universidad de Chile, Santiago, Chile; 2 Bone Biology Laboratory, School of Pharmaceutical Sciences, Universidade de São Paulo, Ribeirão Preto, Brazil; 3 Cellular and Molecular Immunology Laboratory, Center for Biomedical Research and Innovation (CiiB), Universidad de Los Andes, Santiago, Chile; 4 Center of Interventional Medicine for Precision and Advanced Cellular Therapy (IMPACT), Santiago, Chile; 5 School of Medical Technology, Faculty of Sciences, Pontificia Universidad Católica de Valparaíso, Valparaíso, Chile; 6 School of Medical Technology, Faculty of Sciences, Universidad de San Sebastián, Santiago, Chile; 7 Department of Growth, Development and Public Health, Faculty of Dentistry, Universidad de Los Andes, Santiago, Chile; 8 Department of Conservative Dentistry, Faculty of Dentistry, Universidad de Chile, Santiago, Chile

**Keywords:** CD4-positive T-Lymphocyte, Cellular senescence, p38 MAPK, Mitophagy, SASP

## Abstract

**Background:**

In several diseases, senescent T lymphocytes increase in number and release a senescence-associated secretory phenotype (SASP) with inflammatory and osteoclastogenic potential, favoring inflammation and bone loss. It is well known that the activation of p38 mitogen-activated protein kinase (p38 MAPK) orchestrates senescence in CD8^+^ T lymphocytes. However, p38 MAPK contribution to CD4^+^ T lymphocyte senescence remains less comprehensively characterized and warrants further investigation. This study investigates the contribution of p38 MAPK to senescence in CD4^+^ T lymphocytes, focusing on mitochondrial dysfunction and SASP production to elucidate their pathological potential.

**Results:**

Splenic CD4^+^ T lymphocytes isolated from wild-type C57BL/6 mice were subjected to subcytotoxic oxidative stress by H_2_O_2_ exposure to generate stress-induced premature senescence. H_2_O_2_-exposed CD4^+^ T lymphocytes exhibited hallmark features of senescence, including increased cell size, reduced cell proliferation, and upregulation of the cell cycle regulators p16^Ink4a^ and p21^Cip1^. Additionally, these cells displayed defective mitophagy, accumulation of dysfunctional mitochondria, and a SASP enriched in Th17-associated cytokines. In senescence-induced CD4^+^ T lymphocytes, an increase in the expression of phospho-p38 MAPK was also detected. The senescence changes were reversed when p38 MAPK was blocked using the specific inhibitor BIRB-796. In particular, neutralizing p38 MAPK reduced mitochondrial dysfunction and Th17-type SASP production, demonstrating its critical role in driving these senescent traits in CD4^+^ T lymphocytes. These findings ratify the involvement of p38 MAPK as a central regulator of CD4^+^ T lymphocyte senescence, particularly concerning the accumulation of dysfunctional mitochondria and pro-inflammatory SASP production.

**Conclusions:**

This study provides critical insights into immune aging mechanisms in CD4^+^ T lymphocytes and underscores the therapeutic potential of targeting p38 MAPK to mitigate senescence-driven inflammatory diseases.

**Supplementary Information:**

The online version contains supplementary material available at 10.1186/s12979-025-00526-8.

## Background

Cellular senescence is a state of stable proliferative arrest accompanied by phenotypic and functional changes in response to unresolved DNA damage [1]. Particularly, immune-senescence encompasses structural and functional changes in the immune system that increase susceptibility to infections, autoimmunity, and chronic inflammatory diseases such as cardiovascular disorders, neurodegeneration, cancer, and type 2 diabetes [2]. T lymphocytes are particularly prone to senescence among immune cells due to proliferative stress during clonal expansion, leading to replicative senescence [3, 4]. Moreover, T lymphocytes are susceptible to senescence when exposed to oxidative stress, persistent microbial antigenic load, and chronic inflammatory conditions, triggering stress-induced premature senescence (SIPS) [3, 4]. Senescent T lymphocytes contribute to chronic inflammation and persistent infections by producing senescence-associated secretory phenotype (SASP) mediators [5, 6].

In several non-immune cells, the activation of p38 mitogen-activated protein kinase (p38 MAPK) plays a central role in initiating the senescence response and establishing the senescent phenotype. Specifically, p38 MAPK (a) stabilizes and activates p53, a key regulator of the DNA damage response [7]; (b) induces the expression of p16^Ink4a^ and p21^Cip1^, which inhibit cyclin-CDK complexes to enforce cell cycle arrest [8, 9]; (c) inhibits autophagy by negatively regulating the p38IP-mAtg9 interaction [10]; (d) enhances mitochondrial reactive oxygen species (ROS) production and modulates antioxidant enzyme expression, disrupting redox balance and leading to the accumulation of dysfunctional mitochondria [11]; and (e) regulates the release of SASP [12]. These pathways collectively underscore the multifaceted role of p38 MAPK in shaping the senescent phenotype and mediating its functional consequences.

In the immune context, p38 MAPK governs SASP release and drives cellular senescence in CD8^+^ T lymphocytes by inhibiting autophagy, leading to dysfunctional mitochondria accumulation and increased ROS production [13, 14]. Age-associated CD4^+^ T lymphocytes also exhibit impaired autophagy, mitochondrial dysfunction, and elevated levels of ROS, which activate STAT-3 phosphorylation and promote the production of a Th17-type cytokine pattern [15]. However, whether p38 MAPK activation underlies these mitochondrial and secretory alterations in senescent CD4^+^ T lymphocytes remains to be elucidated. This study aimed to investigate the role of p38 MAPK in driving the senescent phenotype in CD4^+^ T lymphocytes, focusing on its regulation of dysfunctional mitochondria accumulation and the release of Th17-type SASP factors with inflammatory potential.

## Methods

### Generation of senescent CD4^+^ T lymphocytes

CD4^+^ T lymphocytes were purified from the spleen of 6- to 8-week-old wild-type C57BL/6 mice. Spleens were mechanically disrupted using the plunger of a sterile syringe and passed through a 40 μm cell strainer to obtain a single-cell suspension. CD4⁺ T lymphocytes were then purified using the CD4⁺ T Cell Isolation Kit (Miltenyi Biotec), according to the manufacturer’s instructions. Immediately after purification, cells were exposed to subcytotoxic oxidative stress by incubation with 400 µM H_2_O_2_ for 1 h [16–18]. Following treatment, cells were washed twice with PBS containing 5% fetal bovine serum (FBS, Thermo Fisher), resuspended at a concentration of 1 × 10^6^ cells/ml, and cultured in 96-well plates in RPMI-1640 medium supplemented with 1% penicillin/streptomycin, 10% FBS, and 20 ng/ml recombinant mouse IL-2 (R&D Systems) for 4 days for downstream analyses.

### Cell viability

To assess the viability of H₂O₂-exposed CD4⁺ T lymphocytes, cells were incubated with Alamar Blue™ reagent (Invitrogen) for 4 h at 37 °C in the dark. Absorbance was measured at 600 nm using a Synergy HTX microplate reader (BioTek, Agilent Technologies).

### Cell proliferation

Cell proliferation was assessed using the CellTrace™ Violet Cell Proliferation Kit (Invitrogen), following the manufacturer’s instructions. CD4⁺ T lymphocytes were labeled with CellTrace™ Violet for 20 min at 37 °C before H_2_O_2_ exposure. Subsequently, cells were stimulated using a T Cell Expansion Kit containing anti-CD3ε/anti-CD28-coated beads (Miltenyi Biotec) to induce proliferation. After 4 days of culture, cell divisions were analyzed by flow cytometry. To evaluate proliferation exclusively in viable cells, H_2_O_2_-exposed CD4⁺ T lymphocytes were stained with Zombie NIR viability dye (BioLegend) for 20 min at room temperature. Then, cells were labeled with a FITC-conjugated anti-CD4 monoclonal antibody (clone RM4-5, BioLegend) for 30 min at 4 °C. The CD25 expression was measured using an APC-conjugated monoclonal anti-CD25 antibody (clone 3C7; BioLegend) to assess cell activation. Data were acquired using an LSR Fortessa X-20 flow cytometer (BD Biosciences) and analyzed with FlowJo software v10.1 (BD Biosciences).

### Expression of p16^Ink4a^ and p21^Cip1^

The mRNA expression levels of the cell cycle inhibitors p16^Ink4a^ and p21^Cip1^ in the H_2_O_2_-exposed CD4^+^ T lymphocytes were analyzed by RT-qPCR. The total cytoplasmic RNA was extracted using the TRIzol™ Plus reagent (Invitrogen), and the first-strand cDNA was synthesized using the iScript™ Reverse Transcription Supermix (Bio-Rad), following the manufacturer’s protocol. Then, 50 ng of cDNA were amplified using gene-specific primers (Table 1) and the KAPA SYBR^®^ Fast qPCR reagent (KAPA Biosystems) in a StepOnePlus™ Real-Time qPCR System (Applied Biosystems). Amplification was performed under the following cycling conditions: initial denaturation at 95 °C for 3 min, followed by 40 cycles of denaturation at 95 °C for 3 s and annealing/extension at 60 °C for 30 s. A melting curve analysis was conducted to confirm the specificity of amplification products: 95 °C for 15 s, 60 °C for 1 min, and 95 °C for 15 s. 18 S rRNA was used as an internal reference gene, and relative mRNA expression levels were calculated using the 2^−ΔΔCt^ method.

**Table 1:** Primers used for mRNA amplifications by RT-qPCR

Target	Primer	Sequence
*p16* ^*Ink4a*^	Forward	AGGACCCCACTACCTTCTCC
	Reverse	TCCCAGCGGTACACAAAGAC
*p21* ^*Cip1*^	Forward	GTACTTCCTCTGCCCTGCTG
	Reverse	AATCTGTCAGGCTGGTCTGC
*MFN2*	Forward	GTGGGCTGGAGACTCATCG
	Reverse	CTCACTGGCGTATTCCACAA
*DRP1*	Forward	AGGTGGCCTTAACACTATTGACA
	Reverse	AGACGCTTAATCTGACGT TTGAC
*RORγt*	Forward	CGCGGAGCAGACACACTTA
	Reverse	CCCTGGACCTCTGTTTTGGC
*Tbx 21*	Forward	GCCAGGGAACCGCTTATATG
	Reverse	GACGATCATCTGGGTCACATTGT
*STAT-4*	Forward	CCAACTCAGACAGCAACT
	Reverse	GCTCTTTGAGCAGGGATGG
*CCL4*	Forward	CCTACCAAGGGTTGATTTTGAG
	Reverse	GACTTGCCGCTCTTCAGTATCT
* 18S rRNA*	Forward	GTAACCCGTTGAACCCCATT
	Reverse	CCATCCAATCGGTAGTAGCG

### Phospho-p38 MAPK detection

The detection of phospho-p38 MAPK in the H_2_O_2_-exposed CD4^+^ T lymphocytes was assessed by Western Blot. Cells were lysed in RIPA buffer supplemented with a phosphatase and protease inhibitor cocktail on ice (Thermo Fisher). Lysates were clarified by centrifugation, and protein concentration was measured using a BCA Pierce protein assay kit (Thermo Fisher). Then, samples were added to 4X Laemli loading buffer (Bio-Rad), and the amount of protein was normalized at 20 µg. Proteins were subjected to separation in a 10% SDS-PAGE gel, and immunoblotting was carried out in a 0.2 μm nitrocellulose membrane using a Trans-Blot Turbo system (Bio-Rad). The membrane was incubated with the following primary antibodies: rabbit anti-mouse-phospho-p38 (Cell Signaling Technology), rabbit anti-mouse-p38 (Abcam), and rabbit anti-mouse-β-actin (Santa Cruz Biotechnology) 1:2000, overnight at 4 °C. Then, an HRP-conjugated anti-rabbit-secondary antibody (Invitrogen) 1:2000 was incubated for 1 h at room temperature. The semi-quantitative analysis was performed using the ImageJ/Fiji software (NIH).

### p38 MAPK neutralization

To neutralize the p38 MAPK activity, the H_2_O_2_-exposed CD4^+^ T lymphocytes were cultured for 4 days in the presence of the p38 MAPK-specific inhibitor BIRB-796 500 nM (MedChemExpress), with DMSO used as the vehicle control. To assess the involvement of autophagy, CD4⁺ T lymphocytes were co-treated with BIRB-796 and the autophagy inhibitor Bafilomycin A1 200 nM (Invitrogen) under the same culture conditions. Rapamycin 1 µM (Invitrogen) was included as a positive control for autophagy induction.

### Lysosomal mass and SA-β-Gal activity

Lysosomal mass and senescence-associated β-galactosidase (SA-β-Gal) activity were evaluated to determine whether H_2_O_2_-exposed CD4⁺ T lymphocytes exhibited altered lysosomal/autophagic function. Lysosomal mass was assessed using LysoTracker™ Red DND-99 (Invitrogen), and SA-β-Gal activity was measured with the FastCellular^®^ Senescence Assay Kit (MP Biomedicals), following the manufacturer’s instructions. Flow cytometry analysis was performed as described above, incorporating the Zombie NIR viability dye (BioLegend) to exclude dead cells and an anti-CD4 monoclonal antibody conjugated to either APC or FITC (clone RM4-5; BioLegend) for cell identification.

### Cathepsin B activity

The Cathepsin B activity in the H_2_O_2_-exposed CD4^+^ T lymphocytes was also analyzed. Cells were incubated with the Magic Red Cathepsin B Assay kit (ImmunoChemistry Technologies) for 4 h at 37 °C, according to the manufacturer’s recommendations. The fluorescence intensity was measured at 600 nm using a Synergy HTX microplate reader (BioTek, Agilent Technologies) and normalized to the cell number in each experimental condition.

### Mitochondria and lysosomes co-localization

To further assess whether H_2_O_2_-exposed CD4⁺ T lymphocytes exhibit impaired mitophagy, the co-localization area between mitochondria and lysosomes was analyzed by immunofluorescence using the CytoPainter™ Lysosome/Mitochondria/Nuclear Staining Kit (Abcam) in live cells, following the manufacturer’s instructions. Images were acquired using a time-lapse confocal microscope (Nikon) and analyzed with ImageJ/Fiji software (NIH). To complement this analysis, additional immunostaining was performed on fixed cells. CD4⁺ T lymphocytes were allowed to adhere to glass coverslips by gravity sedimentation, then fixed with 4% paraformaldehyde for 20 min at room temperature. Cells were permeabilized with 0.1% Triton X-100 for 20 min and blocked with 3% bovine serum albumin (BSA) in PBS for 1 h at room temperature [19, 20]. Primary antibodies against LAMP1 (clone 1D4B; BD Pharmingen) and HSP70 (clone 13D3; Invitrogen) were incubated overnight at 4 °C. After washing, cells were incubated for 1 h at room temperature, protected from light, with Alexa Fluor^®^-conjugated secondary antibodies (Life Technologies). Coverslips were mounted using an anti-fade mounting medium containing DAPI (Abcam) and allowed to dry overnight. Fluorescence images were acquired using a Zeiss LSM 880 laser scanning confocal microscope equipped with Airyscan detection and a 63x oil immersion objective. Mitophagy was evaluated by quantifying the degree of co-localization between mitochondria (HSP70) and lysosomes (LAMP1), using Manders’ overlap coefficients, calculated with the JACoP plugin in ImageJ [21, 22].

### PINK1 assay by flow cytometry

PINK1 expression in H_2_O_2_-exposed CD4⁺ T lymphocytes was assessed by flow cytometry. To exclude non-viable cells and identify CD4⁺ T lymphocytes, samples were stained with Zombie NIR viability dye (BioLegend) and a PerCP-Cy5.5-conjugated anti-CD4 monoclonal antibody (clone GK1.5; BioLegend). Cells were then fixed and permeabilized using a fixation/permeabilization buffer set (Invitrogen), followed by incubation with a FITC-conjugated anti-PINK1 polyclonal antibody (Novus Biologicals) for 1 h at room temperature. Flow cytometry data acquisition and analysis were performed as described above.

### Mitochondrial mass, mitochondrial membrane potential, and ROS production

In the H_2_O_2_-exposed CD4^+^ T lymphocytes, mitochondrial mass was assessed using a MitoTracker™ GreenFM kit (Invitrogen) following the manufacturer’s protocol. The mitochondrial membrane potential was measured using a tetramethylrhodamine ethyl ester perchlorate (TMRE) reagent (Invitrogen), using carbonyl cyanide m-chlorophenylhydrazone (CCCP) reagent (Invitrogen) to disrupt the mitochondrial membrane potential. Mitochondrial ROS production was analyzed using a MitoSox™ Red reagent (Invitrogen). In addition, cytoplasmic ROS production was analyzed using a CellRox™ Green (Invitrogen) or 2′,7′-dichlorodihydrofluorescein diacetate reagent (H2DCFDA, Invitrogen), according to the manufacturer’s instructions. Flow cytometry analysis was performed using Zombie NIR viability dye (BioLegend) and an anti-CD4 monoclonal antibody conjugated to either APC or FITC (clone RM4-5; BioLegend) for cell identification.

### Mitochondrial morphology by transmission electron microscopy

Mitochondrial number, area, and roundness were analyzed by transmission electron microscopy (TEM) in the H_2_O_2_-exposed CD4^+^ T lymphocytes. Cells were fixed in 2.5% glutaraldehyde (pH 7.2) and post-fixed with 1% osmium tetroxide (OsO₄). Then, cells were dehydrated through a graded acetone-water series and embedded in epoxy resin. Ultrathin sections were prepared using a diamond knife, mounted onto 300-mesh copper grids, and stained with 2% uranyl acetate, followed by lead citrate. Images were acquired using a Talos F200C G2 transmission electron microscope (Thermo Fisher) equipped with a Ceta 16 M CMOS camera (Thermo Fisher). Mitochondrial morphology was assessed by manually outlining individual mitochondria, and parameters were quantified on a per-cell basis.

### Mitochondrial fusion/fission analysis by RT-qPCR

To investigate alterations in mitochondrial dynamics, the mRNA expression levels of MFN2 (a key regulator of mitochondrial fusion) and DRP1 (a key regulator of mitochondrial fission) were evaluated by RT-qPCR using gene-specific primers (Table 1), following the protocol described above.

### SASP production

SASP production was assessed in senescent and non-senescent CD4⁺ T lymphocytes under both unstimulated (basal) and stimulated conditions, the latter involving anti-CD3ε/anti-CD28-coated beads (Miltenyi Biotec). Cell culture supernatants were collected, filtered through a 0.22 μm membrane filter (Merck), and analyzed using the Milliplex Mouse Th17 Magnetic Bead ELISA Kit (Merck), following the manufacturer’s instructions. Cytokine concentrations were normalized to the number of cells in each experimental condition. Additionally, intracellular IL-17A expression was evaluated in H_2_O_2_-exposed CD4⁺ T lymphocytes. Unstimulated cells were induced with 50 ng/mL PMA and 5 µg/mL ionomycin (Sigma-Aldrich) in the presence of Brefeldin A (BD Biosciences). After stimulation, cells were fixed and permeabilized using a fixation/permeabilization buffer set (Invitrogen), and then stained overnight at 4 °C with a PerCP-conjugated anti-IL-17A monoclonal antibody (clone TC11-18H10.1; BioLegend). Flow cytometry was performed using Zombie NIR viability dye (BioLegend) and a FITC-conjugated anti-CD4 monoclonal antibody (clone RM4-5; BioLegend) to identify viable CD4⁺ T cells. To further characterize the SASP profile, RORγt, Tbx 21, STAT-4, and CCL4 mRNA expression levels were quantified by RT-qPCR using gene-specific primers (Table 1), as described above.

### Statistical analysis

Statistical analyses were performed using GraphPad Prism version 8.0.1 (Dotmatics). Data normality was assessed using the Shapiro-Wilk test. Depending on data distribution, the unpaired Student’s t-test or the Mann-Whitney U test was used to compare two groups. For comparisons involving more than two groups, one-way ANOVA followed by Tukey’s post hoc test was applied. Statistical significance was considered when α < 0.05.

## Results

### Induction and characterization of the senescent CD4^+^ T lymphocytes

**Fig. 1:**
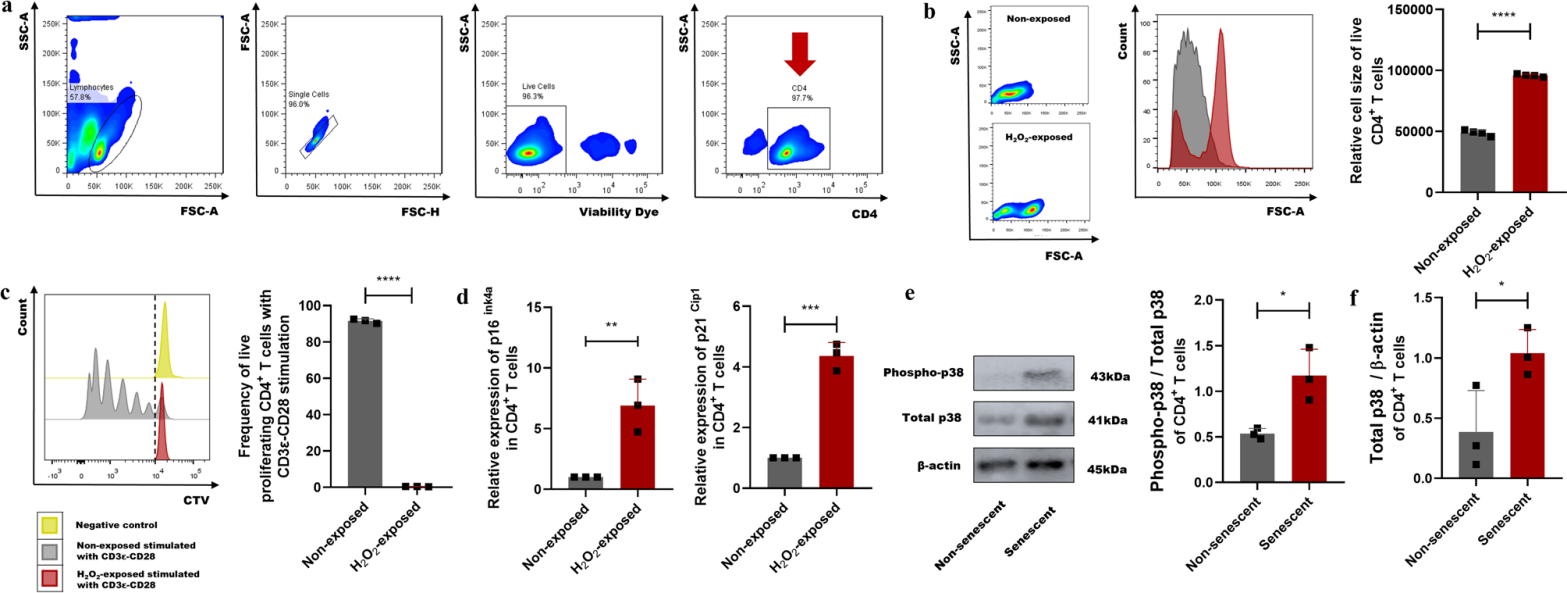
Characterization of the senescent CD4^+^ T lymphocytes. (**a**) Flow cytometry gating strategy used to determine the purity of viable CD4^+^ T lymphocytes isolated from the spleen of wild-type C57 BL/6 mice. The following sequential gating strategy steps were used: First, cells were physically selected according to their cell size (FSC-A) and internal cell complexity (SSC-A) parameters. Second, only single cells were selected according to their FSC-H and FSC-A characteristics, discarding fused cells as duplets or triplets. Third, dead cells were excluded. Finally, the viable CD4^+^ T cells were selected for analysis. This cell characterization was performed separately for each animal. **(b)** Flow cytometry histogram and bar plot show the cell size quantification of viable CD4^+^ T lymphocytes exposed or non-exposed to oxidative stress (H_2_O_2_). **(c)** Flow cytometry histogram and bar plot show the frequency of proliferating CD4^+^ lymphocytes. H_2_O_2_-exposed CD4^+^ T cells stimulated with CD3ε-CD28 (red) were compared with non-exposed CD4^+^ T cells stimulated with CD3ε-CD28 (gray). Non-exposed CD4^+^ T cells without CD3ε-CD28 stimulation were used as negative control (yellow). The dotted line indicates the reference point used to quantify the proliferating cells. **(d)** Relative quantification of the mRNA expression of the cell cycle inhibitors p16^Ink4a^ and p21^Cip1^ in H_2_O_2_-exposed or non-exposed CD4^+^ T cells determined by RT-qPCR. **(e)** Western blot immunodetection of phospho-p38, total p38, and β-actin and quantification of the phospho-p38/total p38 band density in senescent and non-senescent CD4^+^ T lymphocytes. **(f)** Quantification of total p38/β-actin band density in senescent and non-senescent CD4^+^ T lymphocytes. Data are expressed as mean ± SD. Statistical analysis was performed using the unpaired Student’s t-test. *p < 0.05, **p < 0.01, ***p < 0.001, ****p < 0.0001

In the present study, CD4^+^ T lymphocytes were isolated from the spleen of wild-type C57BL/6 mice (Fig. 1a) and immediately subjected to subcytotoxic oxidative stress to induce SIPS-type cellular senescence. CD4^+^ T lymphocytes were treated with 400 µM H_2_O_2_ for 1 h to induce senescence since H_2_O_2_ is an oxidative agent commonly used to induce cellular senescence in vitro [16–18]. Indeed, H_2_O_2_ generates ROS that damage DNA and other macromolecules, triggering a senescent response [16]. Senescence of CD4^+^ T lymphocytes was confirmed 4 days after H_2_O_2_ exposure by analyzing cell proliferation, cell size, and the expression of the cell cycle inhibitors p16^Ink4a^ and p21^Cip1^ [1]. High CD4^+^ T lymphocyte viability (> 75%) was obtained after H_2_O_2_ exposure. The viable H_2_O_2_-exposed CD4^+^ T lymphocytes exhibited increased cell size (Fig. 1b), suppressed proliferative activity (Fig. 1c), and significant upregulation of the p16^Ink4a^ and p21^Cip1^ expression (Fig. 1d), which indicate a stable proliferative arrest, a hallmark of cellular senescence.

### Overexpression of phospho-p38 MAPK in the senescent CD4^+^ T lymphocytes

The activation of p38 MAPK is essential to induce the senescent response and establish a senescent phenotype. Indeed, p38 MAPK promotes cell cycle arrest, inhibits autophagy, favors mitochondrial ROS production, increases the accumulation of dysfunctional mitochondria, and regulates SASP release in senescent cells [7, 8, 10–12]. When activated, the p38 MAPK is phosphorylated at threonine 180 and tyrosine 182 residues and is termed phospho-p38. To demonstrate the p38 MAPK activation in the senescent CD4^+^ T lymphocytes, the expression of phospho-p38 MAPK was analyzed (Figure S1). Oxidative stress increased the expression of phospho-p38 MAPK (Fig. 1e) and total-p38 MAPK (Fig. 1f) in CD4^+^ T lymphocytes, demonstrating the activation of p38 MAPK in senescent CD4^+^ T lymphocytes.

### Disruption of mitophagy in the senescent CD4^+^ T lymphocytes

**Fig. 2:**
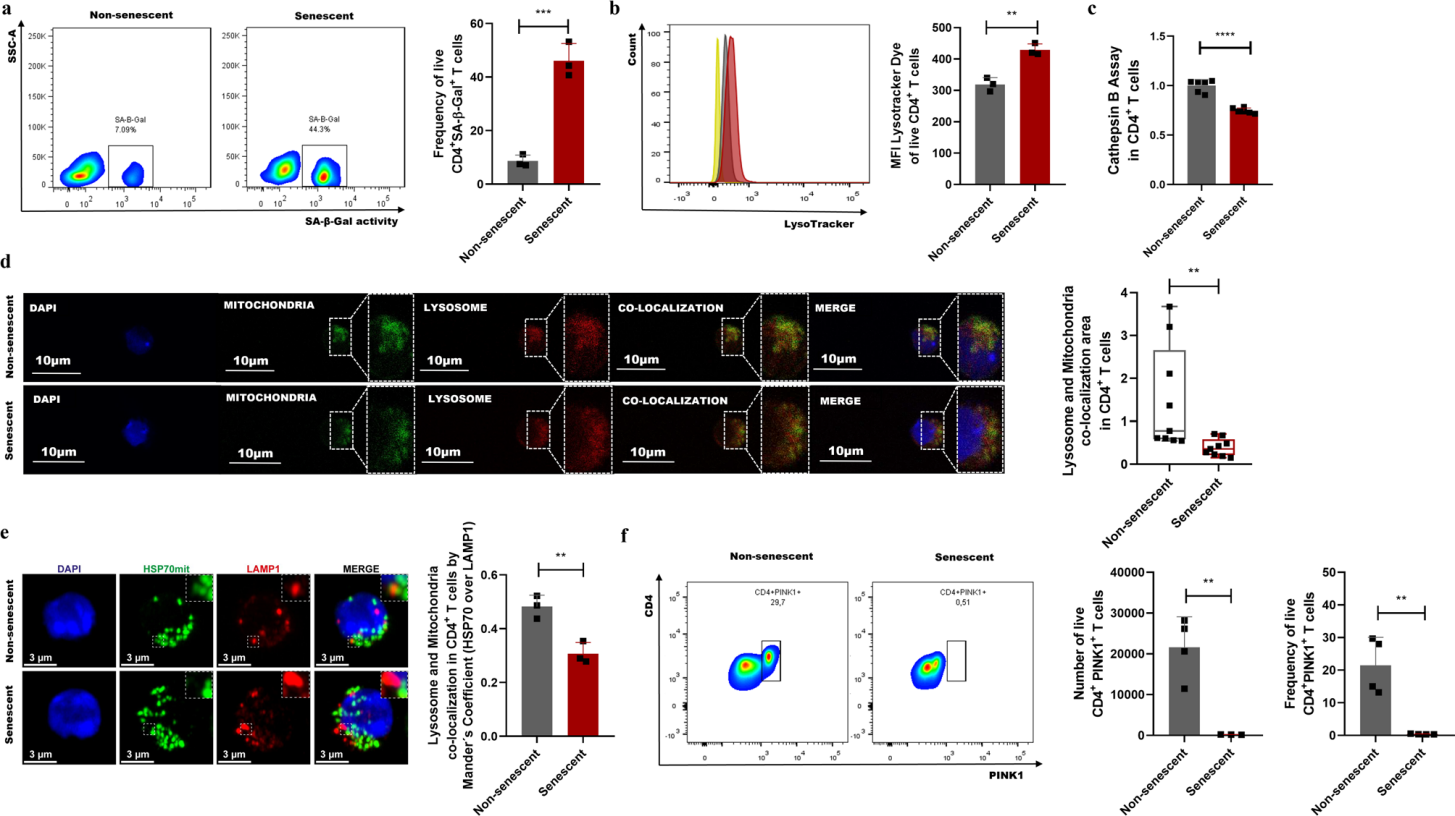
Mitophagy disruption in senescent CD4^+^ T lymphocytes. (**a**) Flow cytometry and bar plots showing the frequency of senescent or non-senescent CD4^+^ T lymphocytes with SA-β-Gal activity (CD4^+^SA-β-Gal^+^ T cells). **(b)** Flow cytometry histogram and bar plot showing the LysoTracker dye MFI in senescent or non-senescent CD4^+^ T lymphocytes. The histogram red peak corresponds to senescent CD4^+^ T lymphocytes, the grey peak to non-senescent CD4^+^ T lymphocytes, and the yellow peak to the negative control (LysoTracker dye MFO). **(c)** Quantification of the activity of Cathepsin B, used as a representative lysosomal enzyme, in senescent and non-senescent CD4^+^ T lymphocytes. **(d)** Representative immunofluorescence images and quantification of the mitochondria and lysosome co-localization area in live senescent and non-senescent CD4^+^ T lymphocytes. Immunofluorescence images show the cell nuclei stained with DAPI (blue), mitochondria (green), lysosomes (red), and area of mitochondria and lysosome co-localization (yellow) in the analyzed CD4^+^ T cells. The box-and-whisker plot shows the median, first and third quartiles as boxes, and 10th and 90th percentiles as whiskers. **(e)** Representative immunofluorescence images and quantification of the mitochondria and lysosome co-localization degree in fixed senescent and non-senescent CD4^+^ T lymphocytes. Immunofluorescence images show the cell nuclei stained with DAPI (blue), HSP70 (mitochondria, green), LAMP1 (lysosome, red), and degree of HSP70 and LAMP1 co-localization (yellow) using Manders’ overlap coefficients. Data are expressed as mean ± SD. **(f)** Flow cytometry and bar plots showing the number and frequency of senescent and non-senescent CD4^+^ T lymphocytes expressing PINK1 (CD4^+^PINK1^+^ T cells). Data are expressed as mean ± SD. Statistical analysis was performed using the unpaired Student’s t-test (bar plots) or the Mann-Whitney U-test (box-and-whisker plot). **p < 0.01, ***p < 0.001, ****p < 0.0001. MFI, mean fluorescence intensity; FMO, fluorescence minus one

Under physiological conditions, cells remove dysfunctional mitochondria by activating the mitophagy process, a self-degradative mechanism that requires the participation of lysosomes [23, 24]. In senescent cells, however, the lysosome function and mitophagy process are altered, facilitating dysfunctional mitochondria accumulation [25]. To evaluate whether these changes occur in H_2_O_2_-exposed CD4^+^ T lymphocytes, the lysosomal function was first evaluated by determining the SA-β-Gal activity since this enzymatic activity is a hallmark of senescent cells [26]. An increase in the frequency of cells with SA-β-Gal activity was observed in senescent CD4^+^ T lymphocytes compared to non-senescent CD4^+^ T lymphocytes (Fig. 2a). Then, the lysosomal mass and the proteolytic capacity were evaluated. An increase in the mean fluorescence intensity (MFI) of the LysoTracker dye was observed in senescent CD4^+^ T lymphocytes, revealing an increase in lysosomal mass (Fig. 2b). The increased lysosomal mass was associated with decreased Cathepsin B activity in senescent CD4^+^ T lymphocytes compared to those non-senescent (Fig. 2c). The co-localization of mitochondria and lysosomes was then analyzed. Compared to non-senescent cells, a significant reduction in the co-localization between these organelles was observed in senescent CD4^+^ T lymphocytes (Fig. 2d and e). Furthermore, the number and frequency of CD4⁺PINK1⁺ cells decreased in senescent CD4⁺ T lymphocytes (Fig. 2f), indicating impaired mitophagy. These findings collectively demonstrate that lysosomal function and the mitophagy process are compromised in senescent CD4⁺ T lymphocytes.

### Accumulation of dysfunctional mitochondria in the senescent CD4^+^ T lymphocytes

**Fig. 3:**
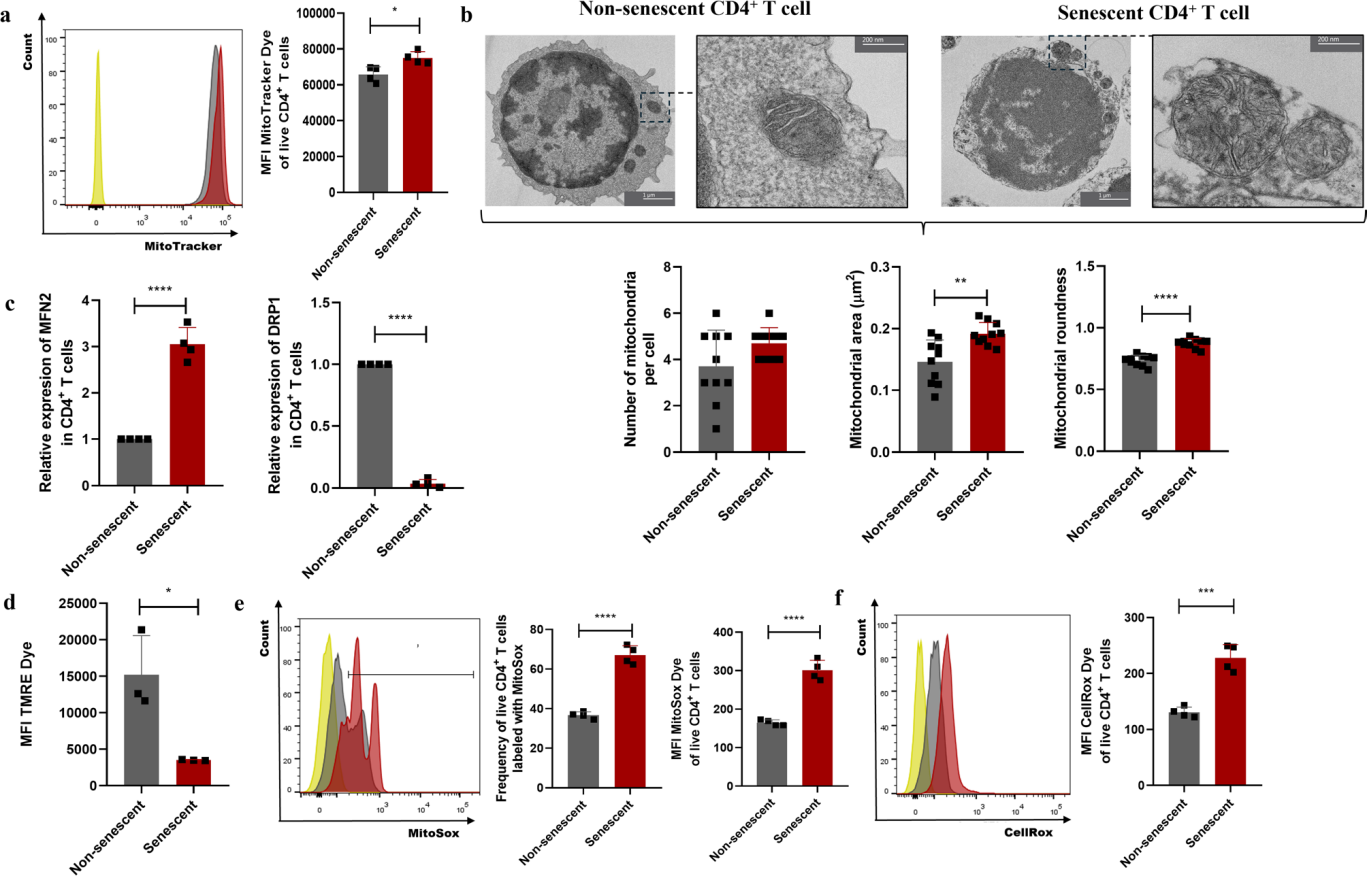
Mitochondrial dysfunction in senescent CD4^+^ T lymphocytes. (**a**) Flow cytometry histogram and bar plot showing the MitoTracker dye MFI in senescent or non-senescent CD4^+^ T lymphocytes. **(b)** Transmission electron microscopy and bar plots showing the mitochondrial number, area, and roundness in senescent or non-senescent CD4^+^ T lymphocytes. **(c)** Relative quantification of the mRNA expression of the fusion/fission markers MFN2 and DRP1 in senescent or non-senescent CD4^+^ T lymphocytes determined by RT-qPCR. **(d)** Bar plot showing the TMRE dye MFI in senescent or non-senescent CD4^+^MitoTraker^+^ T lymphocytes, with the addition of the mitochondrial uncoupler CCCP. **(e)** Flow cytometry histogram and bar plot showing the frequency of MitoSox-positive cells and MitoSox dye MFI in senescent or non-senescent CD4^+^ T lymphocytes. **(f)** Flow cytometry histogram and bar plot showing the CellRox dye MFI in senescent or non-senescent CD4^+^ T lymphocytes. In all flow cytometry histograms, the red peak corresponds to senescent CD4^+^ T lymphocytes, the grey peak to non-senescent CD4^+^ T lymphocytes, and the yellow peak to the negative controls (MitoTracker, MitoSox, and CellRox dye MFO, respectively). Data are expressed as mean ± SD. Statistical analysis was performed using the unpaired Student’s t-test. *p < 0.05, **p < 0.01, ***p < 0.001, ****p < 0.0001. MFI, mean fluorescence intensity; MFO, fluorescence minus one; TMRE, Tetramethylrhodamine ethyl ester perchlorate

Mitochondrial dysfunction is a hallmark of cellular senescence and is key in promoting the senescent phenotype, including the SASP production [25]. In particular, impaired mitophagy leads to the accumulation of dysfunctional mitochondria, resulting in elevated ROS levels that further reinforce the senescent phenotype [26]. To assess mitochondrial status in H_2_O_2_-exposed CD4⁺ T lymphocytes, we analyzed mitochondrial mass, area, number, roundness, membrane potential, and ROS production. Cytoplasmic ROS production was also evaluated. Senescent CD4⁺ T lymphocytes exhibited increased mitochondrial mass, as indicated by higher MitoTracker MFI than non-senescent cells (Fig. 3a). This was accompanied by increased mitochondrial area and roundness, while mitochondrial number remained unchanged (Fig. 3b). Additionally, senescent CD4⁺ T lymphocytes showed upregulation of MFN2 and downregulation of DRP1 (Fig. 3c), suggesting a shift toward mitochondrial fusion. These morphological alterations and changes in the expression of proteins associated with mitochondrial dynamics are consistent with a compromise in mitochondrial quality control mechanisms. A reduced mitochondrial membrane potential was also observed in senescent CD4⁺ T lymphocytes, as revealed by decreased TMRE fluorescence (Fig. 3d). While the loss of mitochondrial membrane potential constitutes a key initial event for PINK1-mediated mitophagy activation, our data revealed a decrease in PINK1 expression in senescent CD4^+^ T lymphocytes (Fig. 2f), suggesting an uncoupling between mitochondrial damage and mitophagy signaling. Regarding mitochondrial ROS, senescent CD4⁺ T lymphocytes displayed an increased frequency of mitochondrial ROS-producing cells and higher ROS production per cell, as shown by the elevated MFI of MitoSOX dye (Fig. 3e). This increase in mitochondrial ROS was associated with greater cytoplasmic ROS levels, evidenced by increased CellROX MFI in senescent CD4⁺ T lymphocytes compared to controls (Fig. 3f). The accumulation of mitochondrial and cytoplasmic ROS reinforces the presence of dysfunctional mitochondria that are not effectively cleared. Altogether, these findings suggest that the accumulation of dysfunctional mitochondria in senescent CD4⁺ T lymphocytes results from impaired mitophagic clearance, contributing to redox imbalance and perpetuation of the senescent phenotype.

### Activation of p38 MAPK drives the accumulation of dysfunctional mitochondria in the senescent CD4^+^ T lymphocytes due to the autophagy disruption

**Fig. 4:**
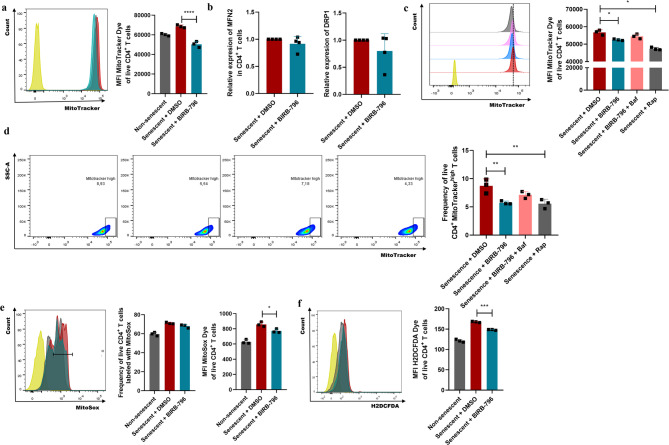
p38 MAPK in dysfunctional mitochondria accumulation and autophagy disruption in senescent CD4^+^ T lymphocytes. (**a**) Flow cytometry histogram and bar plot showing the MitoTracker dye MFI in senescent CD4^+^ T lymphocytes exposed to BIRB-796 or DMSO. Non-senescent CD4^+^ T lymphocytes were used for comparison. **(b)** Relative quantification of the mRNA expression of the fusion/fission markers MFN2 and DRP1 in senescent CD4^+^ T lymphocytes exposed to BIRB-796 or DMSO determined by RT-qPCR. **(c)** Flow cytometry histogram and bar plot showing the MitoTracker dye MFI in senescent CD4^+^ T lymphocytes exposed to BIRB-796, BIRB-796 plus Bafilomycin (autophagy inhibitor), Rapamycin (autophagy inducer), or DMSO. The histogram blue peak corresponds to senescent CD4^+^ T lymphocytes exposed to BIRB-796, the pink peak to senescent CD4^+^ T lymphocytes exposed to BIRB-796 plus Bafilomycin, the grey peak to senescent CD4^+^ T lymphocytes exposed to Rapamycin, the red peak to senescent CD4^+^ T lymphocytes exposed to DMSO, and the yellow peak to the negative control (MitoTracker dye MFO). **(d)** The same analysis described in (c) using the frequency of MitoTracker^high^ senescent CD4^+^ T lymphocytes. **(e)** Flow cytometry histogram and bar plot showing the frequency of MitoSox-positive cells and MitoSox dye MFI in senescent CD4^+^ T lymphocytes exposed to BIRB-796 or DMSO. Non-senescent CD4^+^ T lymphocytes were used for comparison. **(f)** Flow cytometry histogram and bar plot showing the H2DCFDA dye MFI in senescent CD4^+^ T lymphocytes exposed to BIRB-796 or DMSO. Non-senescent CD4^+^ T lymphocytes were used for comparison. In all flow cytometry histograms, the blue peak corresponds to senescent CD4^+^ T lymphocytes exposed to BIRB-796, the red peak to senescent CD4^+^ T lymphocytes exposed to DMSO, the grey peak to non-senescent CD4^+^ T lymphocytes, and the yellow peak to the negative controls (MitoTracker, MitoSox, and H2DCFDA dye MFO, respectively). Data are expressed as mean ± SD. Statistical analysis was performed using the unpaired Student’s t-test, when comparing two groups, or the one-way ANOVA and Tukey post hoc tests, when comparing more than two experimental groups. *p < 0.05, **p < 0.01, ***p < 0.001, ****p < 0.0001. Baf, bafilomycin; DMSO, dimethyl sulfoxide; H2DCFDA, 2′,7′-dichlorodihydrofluorescein diacetate reagents; MFI, mean fluorescence intensity, MFO: fluorescence minus one; Rap, rapamycin

To ascertain whether p38 MAPK activation contributes to the accumulation of dysfunctional mitochondria in the H_2_O_2_-exposed CD4^+^ T lymphocytes, the mitochondrial mass, mitochondrial dynamics, and the mitochondrial and cytoplasmic ROS production were analyzed in the presence of BIRB-796, a specific inhibitor of p38 MAPK [14, 27]. Senescent CD4⁺ T lymphocytes treated with the BIRB-796 inhibitor exhibited a significant reduction in the mitochondrial mass (Fig. 4a). However, no changes were observed in the mRNA expression of MFN2 and DRP1 (Fig. 4b), suggesting that the reduction in mitochondrial mass induced by p38 MAPK inhibition is independent of alterations in mitochondrial dynamics (fusion/fission). To assess whether autophagy mediates the p38 MAPK-dependent senescent changes, senescent CD4^+^ T lymphocytes were co-treated with the p38 MAPK inhibitor BIRB-796 and the autophagy inhibitor Bafilomycin [28], using Rapamycin as an autophagy inducer [29]. BIRB-796 treatment led to a reduction in mitochondrial mass compared to vehicle-treated senescent CD4⁺ T lymphocytes, as evidenced by decreased MitoTracker MFI (Fig. 4c) and lower frequency of MitoTracker^high^ cells (Fig. 4d). In both assays, the extent of mitochondrial mass reduction was comparable to that observed in Rapamycin-treated cells (Fig. 4c and d). However, when autophagy was inhibited with Bafilomycin, the effect of p38 MAPK inhibition on mitochondrial mass was abolished (Fig. 4c and d). These findings suggest that the elimination of mitochondria induced by p38 MAPK inhibition in senescent CD4⁺ T lymphocytes is at least partially mediated by autophagy restoration. Furthermore, BIRB-796 treatment significantly reduced ROS levels in senescent CD4⁺ T lymphocytes compared to vehicle-treated cells, as shown by decreased mitochondrial ROS (MitoSOX MFI; Fig. 4e) and cytoplasmic ROS (H2DCFDA MFI; Fig. 4f). This reduction suggests that the mitochondria targeted for degradation are likely dysfunctional. Taken together, these data suggest that mitochondrial clearance resulting from p38 MAPK inhibition and autophagy restoration may help improve the redox status of senescent CD4⁺ T lymphocytes.

### Activation of p38 MAPK orchestrates the production of a Th17-type SASP in the senescent CD4^+^ T lymphocytes

**Fig. 5:**
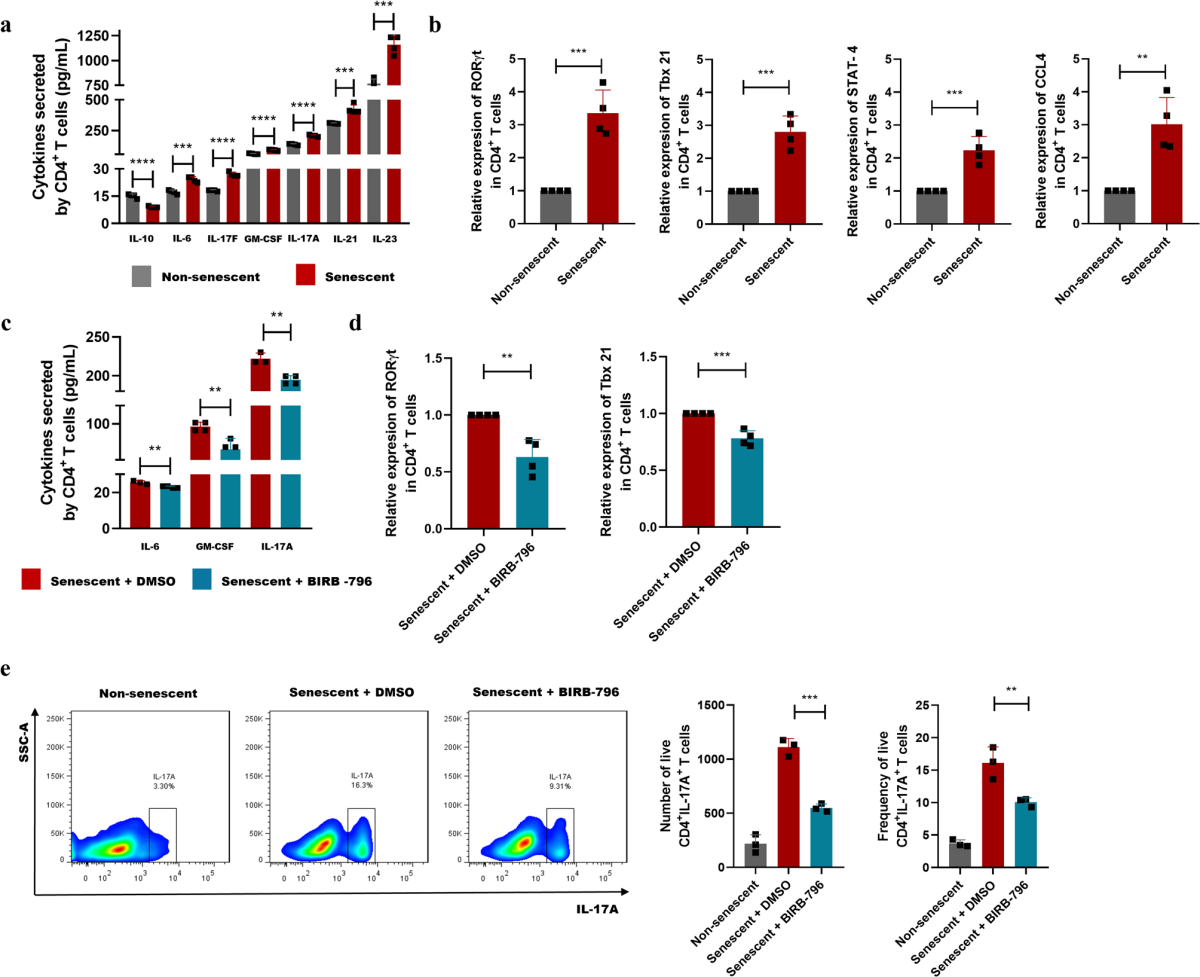
Role of p38 MAPK in the Th17-type SASP release in senescent CD4^+^ T lymphocytes. (**a**) Quantification of the secreted levels of IL-6, IL-10, IL-17A, IL-17F, IL-21, IL-23, and GM-CSF in senescent and non-senescent CD4^+^ T lymphocytes determined by ELISA-Multiplex. **(b)** Relative quantification of the mRNA expression of RORγt, Tbx 21, STAT-4, and CCL4 in senescent and non-senescent CD4^+^ T lymphocytes determined by RT-qPCR. **(c)** Quantification of the secreted levels of IL-6, IL-17A, and GM-CSF in senescent CD4^+^ T lymphocytes exposed to BIRB-796 or DMSO. **(d)** Relative quantification of the mRNA expression of RORγt and Tbx 21 in senescent CD4^+^ T lymphocytes exposed to BIRB-796 or DMSO. **(e)** Flow cytometry and bar plots showing the number and frequency of senescent CD4^+^ T lymphocytes exposed to BIRB-796, exposed to DMSO, and non-senescent CD4^+^ T lymphocytes expressing IL-17A (CD4^+^IL-17A^+^ T cells). Data are expressed as mean ± SD. Statistical analysis was performed using the unpaired Student’s t-test. **p < 0.01, ***p < 0.001, ****p < 0.0001. CCL, chemokine ligand; DMSO, dimethyl sulfoxide; GM-CSF, granulocyte-macrophage colony-stimulating factor; IL, interleukin; ROR, retinoid-related orphan receptor; SASP, senescence-associated secretory phenotype; STAT, signal transducer and activator of transcription; Tbx, T-box transcription factor

The SASP is a hallmark of cellular senescence, characterized by the secretion of a complex mixture of pro-inflammatory cytokines, chemokines, growth factors, and matrix-remodeling enzymes [6]. SASP production is intricately linked to mitochondrial dysfunction and increased ROS production, with the p38 MAPK signaling pathway acting as a central regulator of the transcriptional programs that drive its expression [30]. In the present study, senescent CD4⁺ T lymphocytes produced higher levels of Th17-skewed cytokines, including IL-6, IL-17A, IL-17F, IL-21, IL-23, and GM-CSF, compared to non-senescent cells (Fig. 5a). These cytokines have been directly implicated in the pathogenesis of inflammatory and osteolytic diseases [31–34]. Conversely, senescent CD4⁺ T lymphocytes produced lower levels of the anti-inflammatory cytokine IL-10 (Fig. 5a). At the transcriptional level, senescent CD4⁺ T lymphocytes exhibited increased expression of RORγt, Tbx 21, STAT-4, and CCL4 (Fig. 5b), further supporting their pro-inflammatory and potentially pathogenic profile [35]. Notably, despite their failure to upregulate the activation marker CD25 upon stimulation with anti-CD3ε/anti-CD28 (Figure S2a), senescent CD4⁺ T lymphocytes consistently produced higher levels of Th17-associated cytokines than non-senescent cells under the same stimulatory conditions (Figure S2b). Importantly, pharmacological inhibition of p38 MAPK with BIRB-796 in senescent CD4⁺ T lymphocytes led to a significant reduction in the production of Th17-type mediators (Fig. 5c) and the expression of RORγt and Tbx 21 (Fig. 5d). This p38 MAPK inhibition also markedly decreased the frequency and number of CD4⁺IL-17A⁺ T lymphocytes (Fig. 5e). Together, these results demonstrate that senescent CD4⁺ T lymphocytes produce a pro-inflammatory, Th17-skewed SASP, and this production is orchestrated, at least partly, by the p38 MAPK activation.

## Discussion

**Fig. 6:**
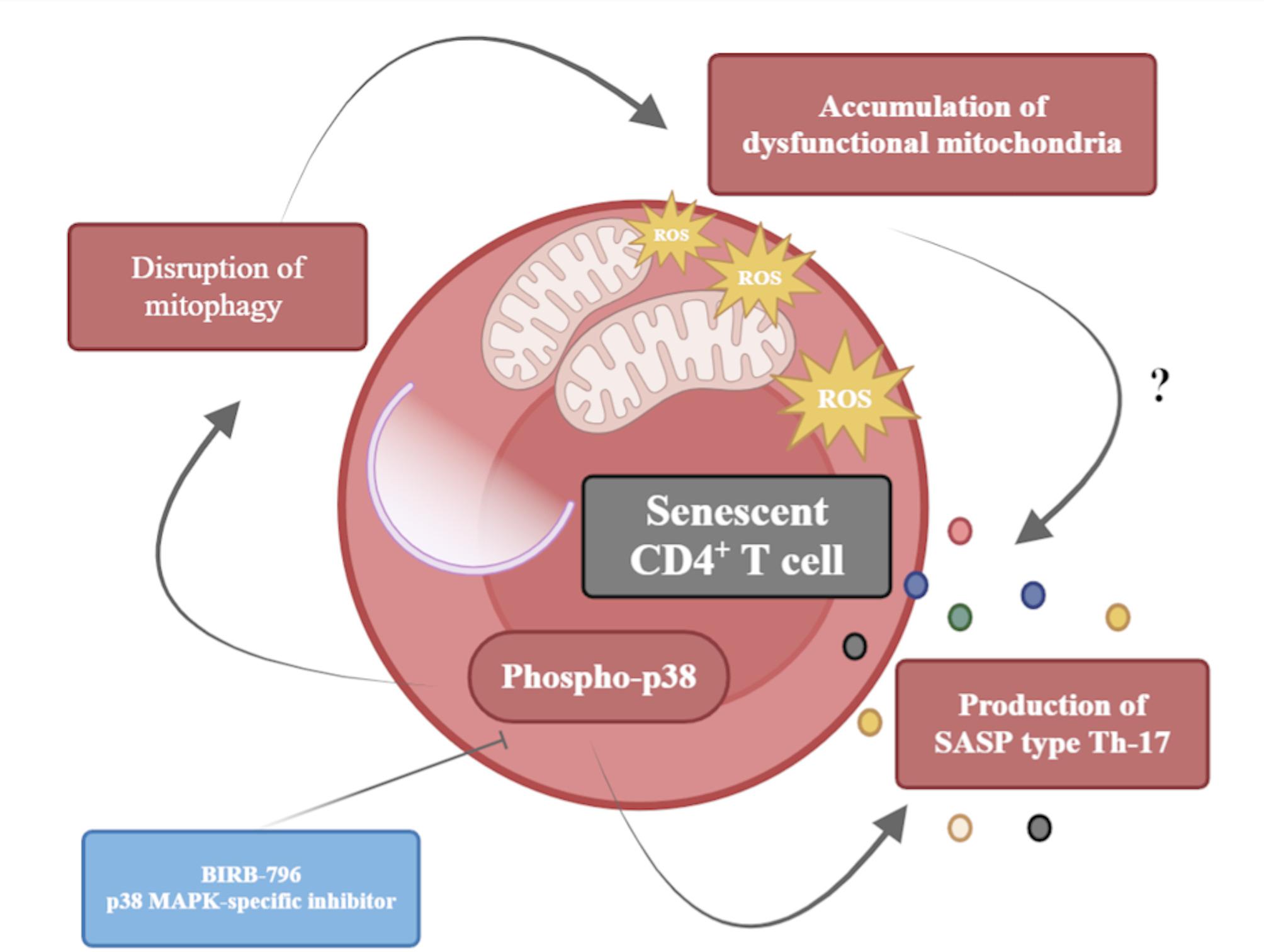
Graphic summary that contextualizes the main findings of the present study. Activation of p38 MAPK drives cellular senescence in CD4^+^ T lymphocytes. Particularly, p38 MAPK activation causes mitophagy disruption, accumulation of dysfunctional mitochondria, and production of an SASP enriched in Th17-type mediators. These cell-senescent phenomena were inhibited when BIRB-796 was used to neutralize the p38 MAPK activation

Among the molecular mechanisms involved in the induction and maintenance of cellular senescence, the p38 MAPK signaling pathway plays a pivotal role. In the immune context, evidence indicates that p38 MAPK activation contributes to T lymphocyte senescence, particularly in CD8⁺ T cells, where it has been closely associated with aging and chronic inflammation [13, 14]. In the present study, we investigated whether a similar mechanism operates in CD4⁺ T lymphocytes subjected to oxidative stress. Our findings demonstrate that p38 MAPK activation is a key mediator of stress-induced senescence in CD4⁺ T lymphocytes, promoting impaired mitophagy, accumulation of dysfunctional mitochondria, and the secretion of a pro-inflammatory SASP enriched in Th17-type cytokines (Fig. 6).

The role of p38 MAPK in cellular senescence has been well established in fibroblasts, the most extensively studied senescent cell type [12]. Senescent fibroblasts display characteristic morphological and functional changes, including increased cell size [36], autophagic-lysosomal dysfunction [17, 26, 37], accumulation of dysfunctional mitochondria [38, 39], and activation of stress-responsive signaling pathways such as p38 MAPK [40]. Similarly, in CD8⁺ T lymphocytes, p38 MAPK has been identified as a central regulator of mitochondrial dysfunction, impaired autophagic-lysosomal function, and SASP production [13, 14]. In CD4⁺ T lymphocytes, p38 MAPK activation has been linked to reduced proliferative capacity and inhibition of telomerase activity [41], suggesting a potential role in promoting senescence. In aged individuals, CD4⁺ T lymphocytes show defective autophagy, mitochondrial dysfunction, and elevated ROS levels, favoring a Th17 differentiation bias [15]. Mitochondrial dysfunction is a critical contributor to SASP generation, as excess ROS derived from damaged mitochondria can trigger the expression and release of pro-inflammatory mediators [30, 42]. Our findings support this framework, showing that oxidative stress-induced senescent CD4⁺ T cells exhibit reduced proliferation, upregulation of senescence-associated cell cycle inhibitors p16^Ink4a^ and p21^Cip1^, increased cell size, impaired mitophagy, accumulation of dysfunctional mitochondria, elevated ROS production, and a Th17-skewed pro-inflammatory SASP. Importantly, these hallmark senescence features largely depended on p38 MAPK activation.

Activation of p38 MAPK generally occurs via two distinct pathways: the classical pathway, which depends on the engagement of the costimulatory molecule CD28, and the alternative pathway, which is activated through sensitization of the T cell receptor (TCR) [43]. In non-senescent CD4⁺ T lymphocytes, these pathways exert functionally distinct effects. Disruption of the classical pathway impairs the differentiation of regulatory T cells, whereas interference with the alternative pathway affects the polarization of pro-inflammatory Th1 and Th17 subsets [44]. Notably, in senescent CD4⁺ T lymphocytes, p38 MAPK activation bypasses these canonical mechanisms and instead occurs in response to intracellular DNA damage, primarily mediated by the ataxia-telangiectasia mutated (ATM) kinase as part of the DNA damage response (DDR) pathway [41, 45]. These findings suggest that senescent CD4⁺ T cells may contribute to inflammation through mechanisms independent of antigenic specificity [46]. Moreover, senescent CD4⁺ T lymphocytes are considered terminally differentiated, with a Th17-skewed phenotype and reduced functional plasticity, a state that may exacerbate their pro-inflammatory potential [47, 48].

Our findings demonstrate that inhibition of p38 MAPK in senescent CD4⁺ T lymphocytes significantly reduces the production of Th17-type cytokines, expression of RORγt and Tbx 21, and frequency and number of IL-17A⁺ T lymphocytes. These results suggest that senescent CD4⁺ T lymphocytes acquire a pro-inflammatory and pro-osteolytic phenotype, potentially contributing to inflammatory and bone-resorptive diseases, which warrants further investigation. The DDR has also been shown to induce mitochondrial dysfunction in senescent CD4⁺ T lymphocytes via the p53-PGC-1α axis [49]. Therefore, p38 MAPK activation and impaired mitophagy may be direct downstream consequences of DDR, functioning as central drivers of a pro-osteolytic SASP enriched in Th17-type cytokines. These insights may be particularly relevant in pathologies such as rheumatoid arthritis and periodontitis, where Th17-mediated inflammation and bone loss are central to disease progression. Nonetheless, further studies are required to elucidate the mechanistic links between DDR, mitochondrial dysfunction, and the Th17-skewed SASP in senescent CD4⁺ T lymphocytes.

While this study provides important insights into the role of p38 MAPK in CD4⁺ T lymphocyte senescence, several limitations should be acknowledged. First, all experiments were conducted in vitro using oxidative stress as the sole senescence-inducing stimulus, which may not fully replicate the complexity of senescence in physiological or pathological contexts. Second, although our findings point to an autophagy-mediated mechanism of mitochondrial degradation following BIRB-796 treatment, further studies assessing the co-localization of mitochondria with mitophagy markers such as PINK1, Parkin, and LC3 are needed to confirm the activation of mitophagy. Third, while our findings implicate p38 MAPK in accumulating dysfunctional mitochondria and SASP production, the upstream signals and potential crosstalk with other regulatory pathways, such as mTOR or NF-κB, remain unexplored. Finally, the exclusive use of oxidative stress to induce senescence may limit the generalizability of our results to other senescence triggers. Future studies incorporating in vivo models and diverse senescence-inducing stimuli will be essential to validate and extend these findings.

Despite these limitations, our results demonstrate pronounced mitophagy disruption in senescent CD4^+^ T lymphocytes. This is evidenced by increased lysosomal mass (Fig. 2a and b), and diminished lysosomal degradative function (Fig. 2c) [50]. These alterations are accompanied by a lack of co-localization between mitochondria and lysosomes (Fig. 2d and e), reflecting a failure in organelle degradation. Furthermore, this mitophagy dysfunction correlates with a marked reduction in PINK1 expression (Fig. 2f), likely secondary to decreased DRP1 levels (Fig. 3c), a protein essential for mitochondrial fission [51]. These findings reveal an important alteration in mitochondrial homeostasis, accumulating structurally abnormal, dysfunctional mitochondria (Fig. 3b and d, and 3e), which contribute to establishing the senescent phenotype [52]. This phenotype is further characterized by the secretion of an SASP enriched in pro-inflammatory mediators (Fig. 5), underscoring the inflammatory potential of senescent CD4⁺ T lymphocytes.

Understanding the mechanisms that drive CD4⁺ T lymphocyte senescence and their contribution to chronic inflammation and bone-resorptive diseases unveils new therapeutic opportunities. Our identification of p38 MAPK as a central regulator of mitochondrial dysfunction, ROS overproduction, and the development of a Th17-skewed SASP underscores its potential as a promising therapeutic target. Pharmacological modulation of p38 MAPK activity could help restore mitochondrial and immune homeostasis, attenuate inflammation, and enhance antigen-specific immune responses. However, additional studies are needed to elucidate these mechanisms further and evaluate the translational potential and safety of targeting this pathway in vivo.

## Conclusions

Our findings shed light on the pivotal role of p38 MAPK activation in senescent CD4^+^ T lymphocytes, driving the accumulation of dysfunctional mitochondria and promoting the release of a Th17-like inflammatory SASP profile. These insights provide a foundation for developing targeted therapies to mitigate the deleterious effects of chronic inflammation and aging-associated diseases, particularly those exacerbated by the accumulation of senescent CD4^+^ T lymphocytes.

## Electronic supplementary material

Below is the link to the electronic supplementary material.

Supplementary Material 1: **Figure S1**. Western Blot immunodetection of phospho-p38 and total-p38 MAPK. (a) Nitrocellulose membrane depicting the sample arrangements: Lanes 1, 2, and 3 correspond to non-senescent CD4+ T lymphocytes, while lanes 4, 5, and 6 correspond to senescent CD4^+^ T lymphocytes. (b) Detection of phospho-p38 MAPK in non-senescent and senescent CD4^+^ T lymphocytes. (c) Detection of total-p38 MAPK in non-senescent and senescent CD4^+^ T lymphocytes. (d) Detection of β-actin as a loading control in non-senescent and senescent CD4^+^ T lymphocytes. All antibody incubations were performed on the same membrane using the stripping method.

Supplementary Material 2: **Figure S2**. Senescent CD4^+^ T lymphocytes exhibit impaired activation (CD25 expression) and enhanced Th17-skewed cytokine production upon anti-CD3ε/anti-CD28 stimulation. (a) Flow cytometry histogram and bar plot show the frequency of proliferating CD4^+^CD25^+^ T lymphocytes stimulated with anti-CD3ε/anti-CD28. (b) Quantification of the secreted levels of IL-6, IL-17A, IL-17F, IL-21, IL-23, and GM-CSF in senescent and non-senescent CD4+ T lymphocytes stimulated with anti-CD3ε/anti-CD28

## Data Availability

All data generated or analyzed during this study were included in the manuscript.

## Abbreviations

ATM: ataxia-telangiectasia mutated
Baf: bafilomycin
CCCP: carbonyl cyanide m-chlorophenylhydrazone
CCL: chemokine ligand
DDR: DNA damage response
DMSO: dimethyl sulfoxide
GM-CSF: granulocyte-macrophage colony-stimulating factor
H2DCFDA: 2′,7′-dichlorodihydrofluorescein diacetate reagents
IL: interleukin
MFI: mean fluorescence intensity
MFO: fluorescence minus one
p38 MAPK: p38 mitogen-activated protein kinase
Rap: rapamycin
ROR: retinoid-related orphan receptor
ROS: reactive oxygen species
SASP: senescence-associated secretory phenotype
SIPS: stress-induced premature senescence
STAT: signal transducer and activator of transcription
Tbx: T-box transcription factor
TCR: T cell receptor
TMRE: tetramethylrhodamine ethyl ester perchlorate
