# Supplementary figures and images for: p38 mitogen-activated protein kinase drives senescence in CD4+ T lymphocytes and increases their pathological potential
Source: Immun Ageing. 2025 Jul 15;22:30. doi: 10.1186/s12979-025-00526-8 (PMC12261694; doi:10.1186/s12979-025-00526-8)

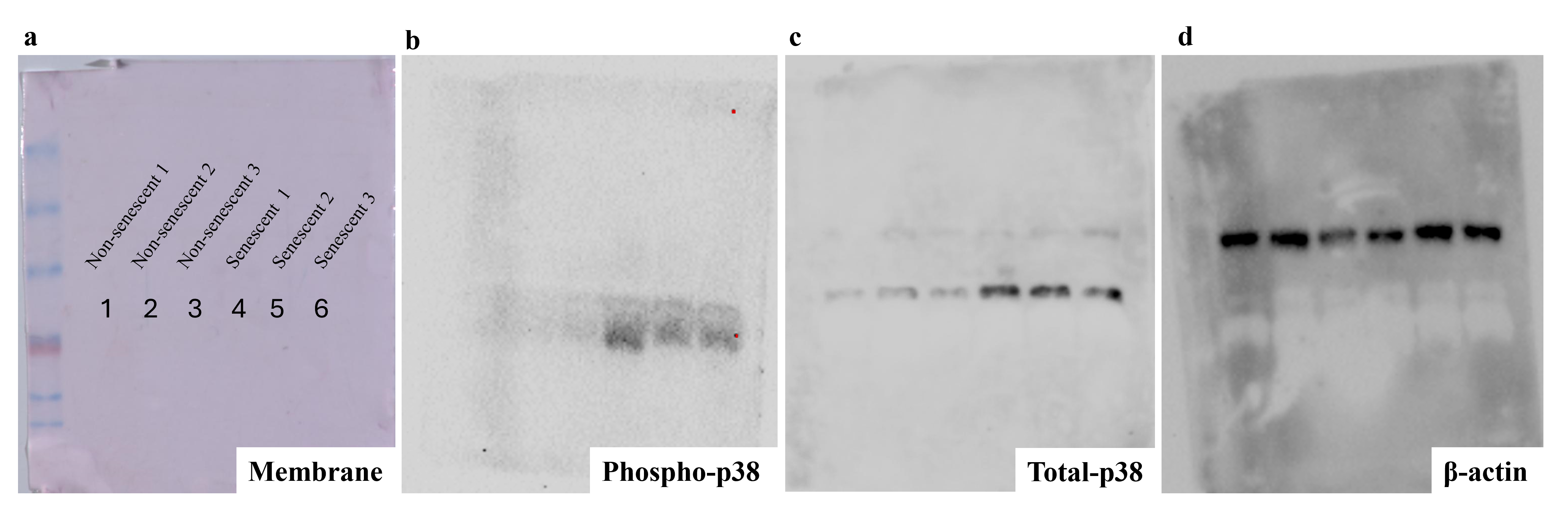

Supplement: Supplementary file 1 — Supplementary Material 1: Figure S1. Western Blot immunodetection of phospho-p38 and total-p38 MAPK. (a) Nitrocellulose membrane depicting the sample arrangements: Lanes 1, 2, and 3 correspond to non-senescent CD4+ T lymphocytes, while lanes 4, 5, and 6 correspond to senescent CD4+ T lymphocytes. (b) Detection of phospho-p38 MAPK in non-senescent and senescent CD4+ T lymphocytes. (c) Detection of total-p38 MAPK in non-senescent and senescent CD4+ T lymphocytes. (d) Detection of β-actin as a loading control in non-senescent and senescent CD4+ T lymphocytes. All antibody incubations were performed on the same membrane using the stripping method. [file 12979_2025_526_MOESM1_ESM.tiff]

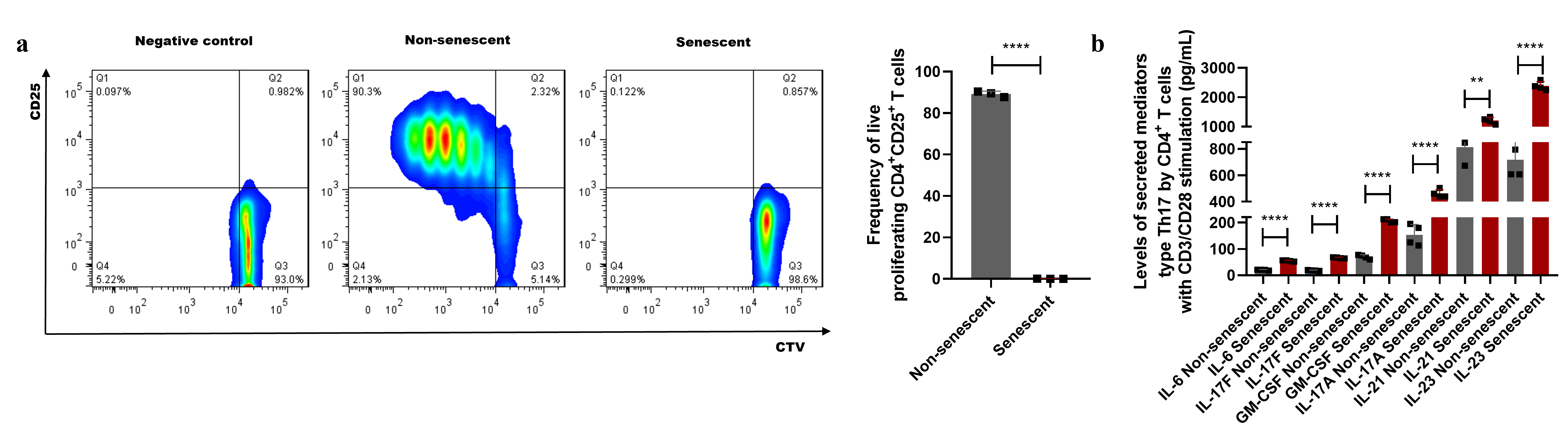

Supplement: Supplementary file 2 — Supplementary Material 2: Figure S2. Senescent CD4+ T lymphocytes exhibit impaired activation (CD25 expression) and enhanced Th17-skewed cytokine production upon anti-CD3ε/anti-CD28 stimulation. (a) Flow cytometry histogram and bar plot show the frequency of proliferating CD4+CD25+ T lymphocytes stimulated with anti-CD3ε/anti-CD28. (b) Quantification of the secreted levels of IL-6, IL-17A, IL-17F, IL-21, IL-23, and GM-CSF in senescent and non-senescent CD4+ T lymphocytes stimulated with anti-CD3ε/anti-CD28 [file 12979_2025_526_MOESM2_ESM.tiff]
